# Supplementary material for: Sialic Acid-Loaded Nanoliposomes with Enhanced Stability and Transdermal Delivery for Synergistic Anti-Aging, Skin Brightening, and Barrier Repair
Source: Pharmaceutics. 2025 Jul 24;17(8):956. doi: 10.3390/pharmaceutics17080956 (PMC12389717; doi:10.3390/pharmaceutics17080956)
Supplement: Supplementary file 1 [file pharmaceutics-17-00956-s001.zip › pharmaceutics-3685751-supplementary.pdf]

Supplementary Materials: Sialic Acid Loaded Nanoliposomes with Enhanced Stability and Transdermal Delivery for Synergistic Anti-Aging, Skin Brightening and Barrier Repair

Fan Yang [1,2], Hua Wang [2], Dan Luo [3], Jun Deng[3], Yawen Hu [2], Zhi Liu [1*], and Wei Liu [1,4*]

1 College of Life Science and Technology, Huazhong University of Science and Technology, Wuhan 430074, China; yangfanfenix@outlook.com
2 Research & Development Center, Mageline Biology Tech Co., Ltd., Wuhan 430000, China; wanghua@mageline.cn; huyawen@mageline.cn
3 Wuhan Bestcarrier Biotechnology Ltd., Wuhan, 430075, PR China; 767698437@qq.com
4 National Engineering Research Center for Nanomedicine, Huazhong University of Science and Technology, Wuhan 430075, China
* Correspondence: zhiliu@hust.edu.cn; wliu@hust.edu.cn

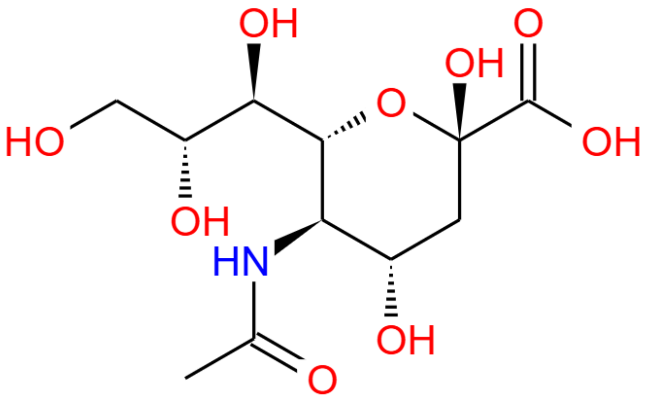

Figure S1. The Chemical structure of SA.

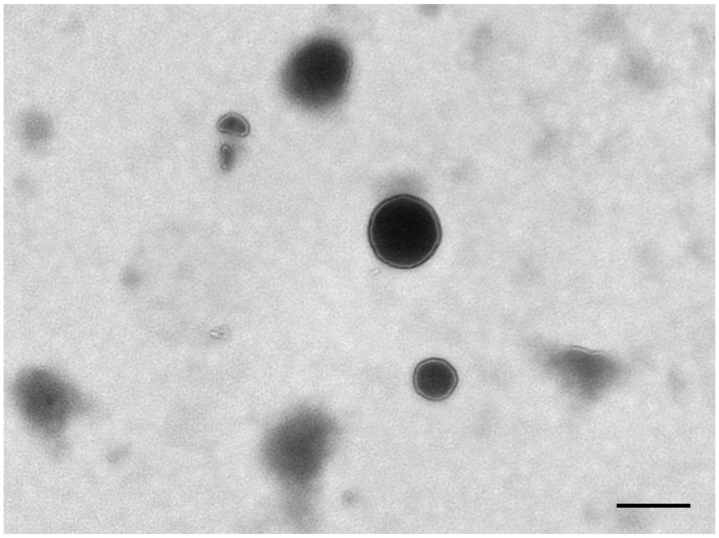

Figure S2. TEM image of the SA-NLPs at high magnification. Scale bar: 50 nm.

Results

			Size (d.n...	% Intensity:	St Dev (d.n...
Z-Average (d.nm):	50.79	Peak 1:	56.39	100.0	18.75
PdI:	0.107	Peak 2:	0.000	0.0	0.000
Intercept:	0.979	Peak 3:	0.000	0.0	0.000
Result quality	Good				

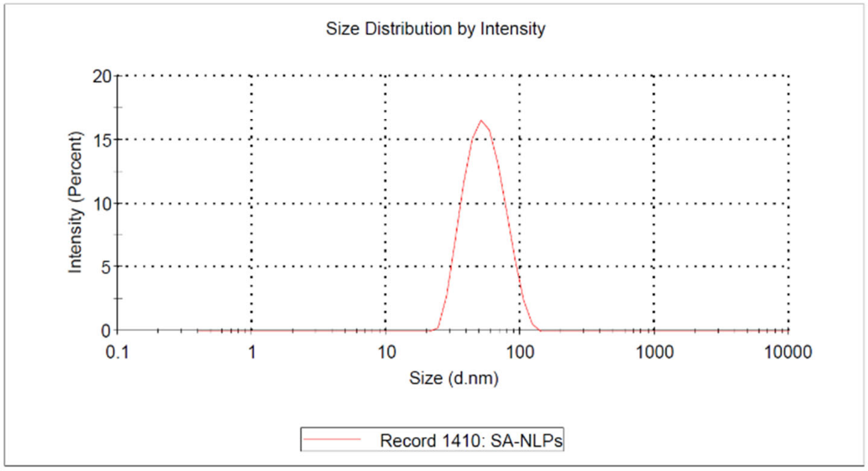

Figure S3. A representative DLS measurement report confirming the quality of the data.

Table S1. Stability of SA-NLPs under different storage conditions over 14 days.

Storage Conditions	Particle Size(nm)	PDI	Zeta Potential (mV)
4°C	50.80±1.2	0.169±0.007	-35.6±0.2
RT	50.72±1.9	0.165±0.009	-33.8±0.5
45°C	51.97±2.4	0.177±0.017	-30.4±0.7
Lighting	50.51±1.4	0.159±0.01	-32.5±0.6
-20°C	50.43±0.8	0.153±0.006	-36.1±0.3

Table S2. Stability of SA-NLPs under different storage conditions over 28 days.

Storage Conditions	Particle Size(nm)	PDI	Zeta Potential (mV)
4°C	52.44±1.6	0.198±0.01	-34.3±0.5
RT	52.71±2.2	0.187±0.014	-32.1±0.7
45°C	53.16±2.9	0.201±0.02	-30.1±0.8
Lighting	51.96±2.0	0.183±0.015	-30.9±0.6
-20°C	52.07±1.4	0.174±0.012	-34.9±0.5

Table S3. IS_{HET} Result of HET-CAM.

No.	Sample	IS_{HET}
1	Normal Saline	0.07
2	0.1% NaOH	10.00
3	Free-SA	0.07
4	SA-NLPs	0.07

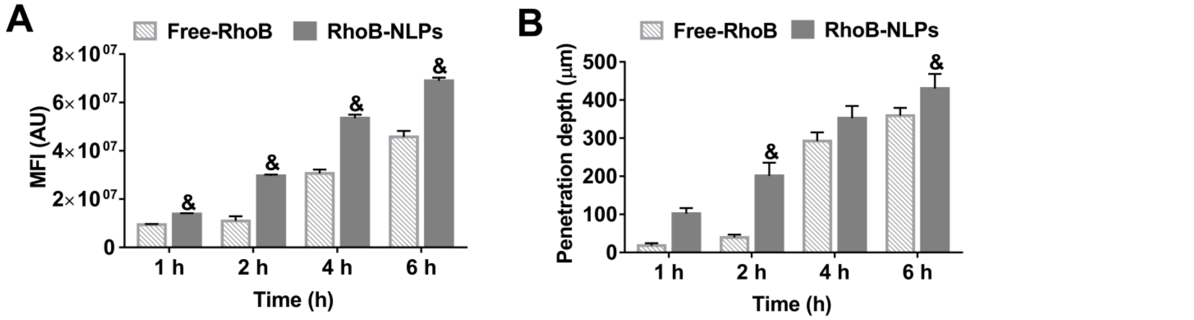

Figure S4. Skin penetration behavior observed via fluorescence microscope with quantitative analysis of (A) MFI and (B) penetration depth. $^{\&}P < 0.05$, $^{\&\&}P < 0.01$ vs Free. Mean±SD, n=3.

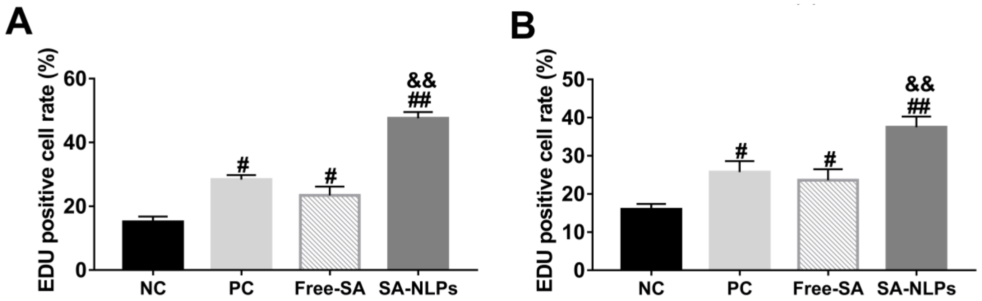

Figure S5. Effect of Free-SA and SA-NLPs on cell proliferation. EdU-positive cell rate of (A) HDF and (B) HaCaT treated with Free-SA and SA-NLPs. $^{\#}P < 0.05$, $^{\#\#}P < 0.01$ vs NC, $^{\&}P < 0.05$, $^{\&\&}P < 0.01$ vs Free, Mean ± SD, n=5.

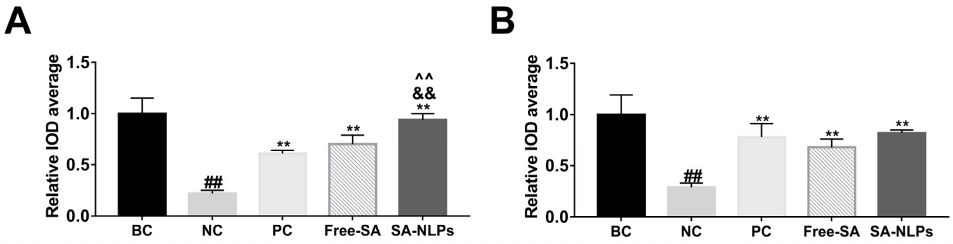

Figure S6. The anti-aging effect was evaluated using Ex-vivo®. The relative value of synthesized (A) COL-I and (B) COL-IV were observed and quantified by fluorescence microscopy. $^{\#}P < 0.05$, $^{\#\#}P < 0.01$ vs BC; $^{*}P < 0.05$, $^{**}P < 0.01$ vs NC; $^{\wedge}P < 0.05$, $^{\wedge\wedge}P < 0.01$ vs NC Mean ± SD, n=3.

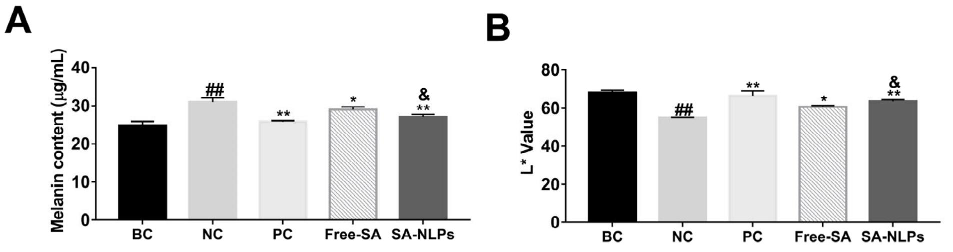

Figure S7. Microscopy images quantified of (A) melanin and (B) L* Value in the MelaKutis® model after UVB exposure at 50 mJ/cm^2 for 24 hours, followed by treatment with Free-SA or SA-NLPs at the same SA concentration. $^{\#}P < 0.05$, $^{\#\#}P < 0.01$ vs BC; $^{*}P < 0.05$, $^{**}P < 0.01$ vs NC; Mean ± SD, n=3.

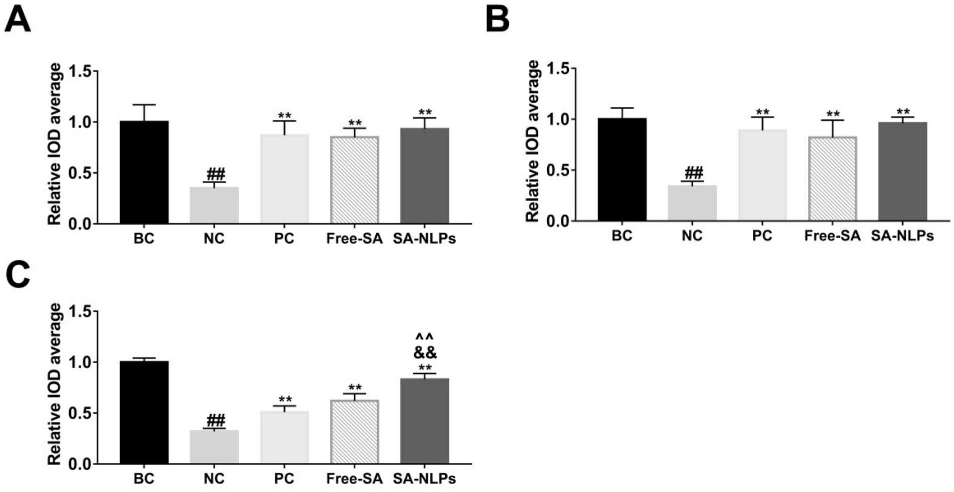

Figure S8. Fluorescent immunization images quantified of (A) FLG, (B) LOR and (C) CLDN1 in the EpiKutis® model treated with 0.2 % SLS, Free-SA or SA-NLPs with the same SA concentration respectively. $^{\#}P < 0.05$, $^{\#\#}P < 0.01$ vs BC; $^{*}P < 0.05$, $^{**}P < 0.01$ vs NC; $^{\wedge}P < 0.05$, $^{\wedge\wedge}P < 0.01$ vs PC; Mean ± SD, n=3.
